# Tooth wear: a cross-sectional investigation of the prevalence and risk factors in Beijing, China

**DOI:** 10.1038/bdjopen.2016.12

**Published:** 2017-01-27

**Authors:** Kan Sun, Wenhui Wang, Xiaozhe Wang, Xiangru Shi, Yan Si, Shuguo Zheng

**Affiliations:** 1 Department of Preventive Dentistry, Peking University School and Hospital of Stomatology, National Engineering Laboratory for Digital and Material Technology of Stomatology, Beijing Key Laboratory of Digital Stomatology, Beijing, China

## Abstract

**Objectives:**

The aim of this study was to evaluate the epidemiology of tooth wear in Beijing and to establish appropriate preventive measures.

**Materials and Methods:**

This cross-sectional analysis involved a questionnaire survey conducted for 1,812 individuals aged 12–74 years in Beijing. Subjects were local residents living in the region for >6 months before the survey. Subjects were evaluated using clinical examinations with the basic erosive wear examination index and a self-administered questionnaire. Statistical analyses were performed using SPSS software.

**Results:**

The prevalence of maxillary tooth wear was 84.9% for the molars, 68.9% for the premolars, 74.1% for the canines and 97% for the incisors. In the mandible, the corresponding prevalence rates were 85.2%, 59.3%, 78.6% and 97.4%, respectively. The occlusal, incisal and cervical surfaces showed more frequent wear compared with the other surfaces. Age, acidic beverages, xerostomia and brushing habits were identified as risk factors for tooth wear (*P*<0.05).

**Conclusions:**

Tooth wear is common in Beijing. Specific preventive measures should be recommended for individuals reporting excessive consumption of fruits and/or acidic beverages, and those with xerostomia. In particular, incisor wear should be carefully monitored in individuals of all age groups.

## Introduction

Tooth wear is a multifactorial disease defined as the loss of hard tissue in the absence of caries or trauma.^1^ It can be classified as attrition, erosion and abrasion.^2^ Attrition is defined as the physiological wear of enamel, dentin or restorations caused by tooth-to-tooth contact.^3^ Erosion is defined as the loss of tooth substance caused by chemical agents, particularly intrinsic or extrinsic acids, with no bacterial involvement. Intrinsic acids originate from gastrointestinal and eating disorders, whereas extrinsic acids are introduced in the oral cavity through the ingestion of foods or beverages, including carbonated soft drinks, fresh fruits and fruit juices. Abrasion is defined as the loss of tooth substance caused by processes involving bio-mechanical friction.^3^

Excessive tooth wear leads to hypersensitivity, pulpitis, periapical periodontitis and pulp necrosis^4^ and can cause serious damage to the oral health of an individual. In the past two decades, the socioeconomic status, dietary habits and lifestyle of individuals have undergone several changes. Furthermore, there is a well-recognised increase in the proportion of aged and elderly individuals in China. Tooth wear has recently emerged as an oral health problem.

There is not much epidemiological data about tooth wear for China. However, studies about erosion, a kind of tooth wear, are relatively complete. In China, the survey of erosion concentrated on the 5–15-year-old children, and its prevalence ranged from 7 to 60%, resulting from different socioeconomic levels, population ages and so on.^5–8^ In China, the studies of abrasion mainly involved the 60–74-year-old population, focusing on the factors of age and brushing habits. Attrition is primarily a physiological wear. However, it is difficult to perform a differential diagnosis according to the pathogen in clinical examination. Instead, we can do some investigation on tooth wear as a multifactorial disease including attrition, erosion and abrasion to understand this non-carious disease. Therefore, we conducted the present study to determine the epidemiological status of tooth wear in Beijing and determine the risk factors in order to gain an understanding of the condition and establish appropriate preventive measures. We derived our patient sample from Beijing, the capital of China, which is densely populated and can provide a good understanding of the tooth wear status in China.

## Materials and Methods

### Study population

The study was approved by the Stomatological Ethics Committee of Chinese Stomatological Association (Approval No. 2014-001). Informed consent was obtained for clinical examination from participants, and for reporting individual patient data. We selected subjects on the basis of five age groups at the time of the survey, namely, 12, 15, 18–35, 36–49 and 50–74 years, and they were evaluated using standardised clinical examinations and a self-administered questionnaire. All subjects were local residents living in the region for >6 months before the survey. The inclusion criteria were as follows: provision of written informed consent after understanding the study purpose and protocols, age 12–74 years, and the willingness to comply with all research procedures and requirements. For children, informed consent was required from the parents or guardian and the child. The exclusion criteria were as follows: previous history of medications for oral diseases, presence of systemic diseases that may affect the integrity of the study data or the safety of the subjects, such as paralysis, leukaemia, epilepsy, depression and other mental disorders, and nursing children. Nursing children were defined as those placed in an institution, organisation or entity for control or protection by the government or government agencies. The children enjoyed the rights prescribed by laws and regulations. These also included children under foster care and those living in a nursing home or institution. Adopted children and children with an official legal guardian were not considered nursing children.

We used a multistage, stratified random sampling method to derive a representative sample. For calculating the sample size (with an estimated prevalence of *P*), we used the formula:

$$n=\frac{4z_{\alpha /2}^{2}\;P(1-P)}{L^{2}}$$

(*P*=30%, 95% confidence interval (CI): 25–35%, *α*=0.05, *Z*_*α*/2_=1.96).

Thus, we calculated that *N*=4×1.96^2^×0.3×0.7/0.1^2^＝322; adding 10%, *N*=354; so we selected 360 individuals in each age group, the total number of subjects being at least 1,810. For this, we first prepared a list of all the districts in Beijing arranged in the order of the Hanyu Pinyin. From these districts, we selected Haidian and Fengtai districts using the simple random sampling method. Then, we listed the communities in these districts in the order of the Pinyin and selected the Agricultural University community, Tsinghua community and Jiaotong University community in Haidian district and the Jiaomen West community, Majiapu community and Dahongmen community in Fengtai district using the random sampling method. From each community, we listed names of people in each age group in the order of the Pinyin and selected 60 individuals belonging to each of the five age groups, with a men:women ratio of 1:1.35. Thus, we selected 1,812 subjects (42.5% men and 57.5% women) for this study.

### Clinical examination

Clinical examinations were conducted by an investigation team including two trained examiners, one field organiser, two recorders and one staff member for disinfection. The required equipment and supplies are as follows: portable dental chairs; portable examination lamps; dental reflectors, tweezers and instrument trash; and other items, such as disinfectant, aseptic cotton buds, one-time plastic cups and pollutant tubs. The examiners had participated in a training programme on tooth wear on 6–7 September 2013 organised by the Chinese Stomatological Association.

All subjects provided written informed consent for use of their data and were willing to answer the questionnaire. All teeth were scored using the criteria shown in Table 1, which were based on the basic erosive wear examination (BEWE) index, which was described in 2008 by Bartlett and was developed to provide a simpler tool for monitoring and recording the severity and progress of erosive tooth wear in general practice.^9^ All four visible surfaces (buccal, cervical, lingual/palatal and occlusal/incisal) of all present teeth were assessed for wear. The example of a check table is shown in Table 2. A total of 28 teeth from 17 to 47 were included for scoring. In the examination, third molars, traumatised or carious teeth, incompletely erupted teeth, teeth with restored surfaces (>25% of the surface) and teeth with appliances were scored ‘8’ and the missing teeth were scored ‘9’. In the data analyses, ‘8’ and ‘9’ were invalid data. All teeth in the maxilla and mandible were divided into incisor, canine, premolar and molar groups. The incisor groups included the central and lateral incisors, the canine groups included the canines, the premolar groups included the first and second premolars, and the molar groups included the first and second molars. A total of eight groups (four maxillary and four mandibular were thus formed).

During the clinical examinations, 5% subjects were reviewed by the same examiner, whereas another 5% were reviewed by another examiner for the evaluation of intra- and inter-examiner reliability. The interval of review was at least 15 min or two subjects. Intra- and inter-examiner reliabilities were evaluated according to the World Health Organization recommendation for a kappa agreement of 0.75 and the actual kappa was 0.85 and 0.80, respectively.

### Questionnaire

Thirty minutes before the clinical examination, a self-administered questionnaire designed on the basis of previous literature and expert opinions was completed by all subjects. The questions pertained to dietary habits, particularly the consumption of acidic foods or beverages; history of drug use; history of gastrointestinal disease; the symptoms of acid or food regurgitation, anorexic or bulimic; bruxism; xerostomia; brushing habits; working environment (acid gas); and socioeconomic status. Examples of the questions are shown in Table 3. In the questionnaire, we used a ladder to indicate the social status, which was divided into 10 steps. From the bottom to the top, the number increased in turn from 1 to 10, symbolising that the status was getting higher in turn. The subjects, according to their own subjective feelings, marked in the corresponding step. The room-mates or family members of the subjects were allowed to help with questions pertaining to bruxism, consumption of fresh fruit and others.

### Data analyses

Analyses were performed on an individual basis. Each tooth in the respective group was assigned a score of 1 if a BEWE score of 2–3 was recorded for any assessed surface. The number of maxillary and mandibular incisors, canines, premolars and molars with a score of 1 was then recorded for each subject to calculate the prevalence rates for the eight groups.

Statistical analyses were performed using SPSS software, version 20 (IBM SPSS Statistics, IBM Corporation, Chicago, IL, USA). The relationship between tooth wear and the questionnaire items was evaluated using a multiple logistic regression model. A *P*-value of <0.05 was considered statistically significant; only those variables with a *P*-value of <0.05 in the final model were considered significant. To determine the significance of differences between groups, *χ*^2^ or Fisher’s exact tests were used, with a *P*-value of <0.05 considered statistically significant.

## Results

**Figure 1:**
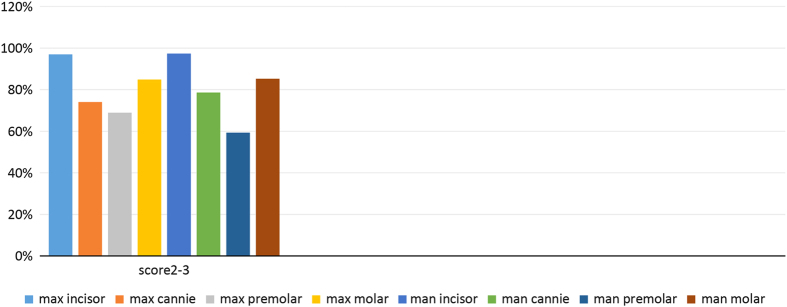
Prevalence of a BEWE score of 2–3 in the eight tooth groups.

**Figure 2:**
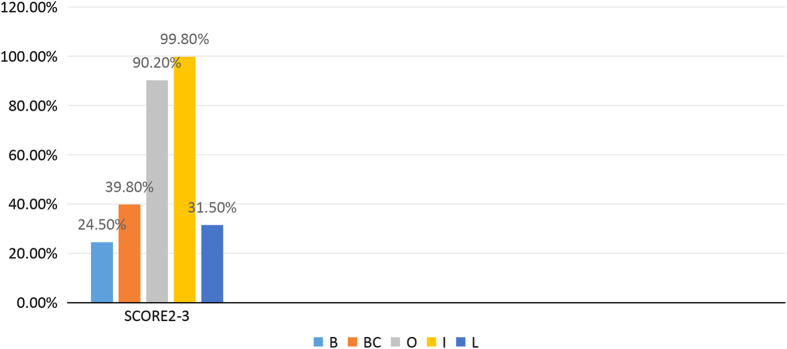
Prevalence of a BEWE score of 2–3 for the different tooth surfaces.

In total, 1,812 subjects (42.5% men and 57.5% women) were included in this study. The prevalence rates for tooth wear (BEWE score, 2–3) in the different groups of teeth are shown in Figure 1. In the maxilla, the rates were 84.9% for the molars, 68.9% for the premolars, 74.1% for the canines and 97.0% for the incisors. In the mandible, the corresponding rates were 85.2%, 59.3%, 78.6% and 97.4%, respectively. The prevalence rates showed no significant difference between maxilla and mandible (*P*>0.05 used *χ*^2^ or Fisher’s exact tests). The prevalence rates of wear for the different surfaces (buccal, cervical, lingual/palatal and occlusal/incisal) are shown in Figure 2. The rates were higher for the occlusal and incisal surfaces than for the other surfaces. The cervical surfaces also showed an increased susceptibility to wear.

**Figure 3:**
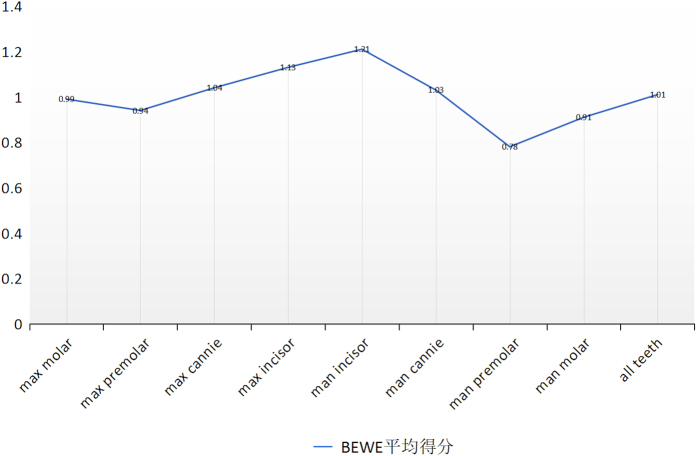
The average of BEWE score for different groups of teeth.

After removing the invalid data and calculating the average of tooth wear of different regions of the teeth, it can be seen from Figure 3 that the average scores of maxilla and mandible incisors are the highest, followed by the canines, molars and premolars in the maxilla and mandible. The average score of whole dentition is 1.01, meaning that the average level of tooth wear in Beijing, China, is level 1, the superficial defects.

**Figure 4:**
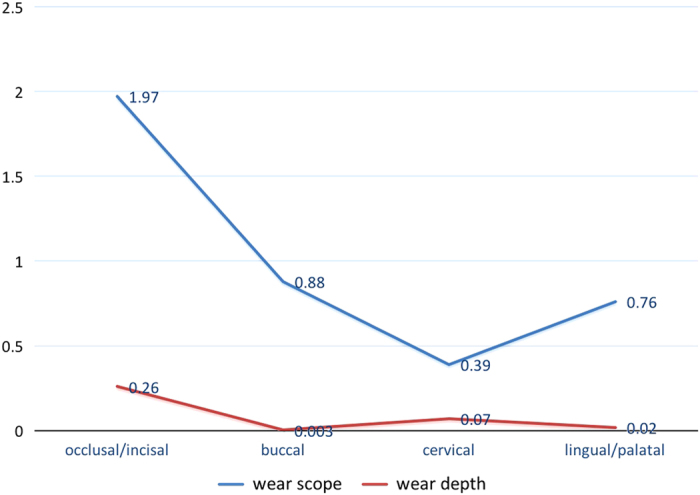
The average of wear scope and wear depth in different surfaces.

We calculated the average score of BEWE and the average of dentin exposure for each inspection site; as shown in Figure 4, in terms of the hard tissue loss of the surface area, the average score of occlusal/incisal surface is the highest, followed by the buccal surface. However, in terms of wear depth, in other words, the situation of dentin exposure, we can see that the average score of occlusal/incisal surface is still the highest, followed by the cervical surface. This is mainly because that BEWE score is focused on the wear area instead of depth and the cervical surface is the predilection site of the wedge-shaped defect.

All participants completed the questionnaire. We used logistic regression to analyse the risk factors and odds ratios to indicate the strength of association. The results revealed that age; fresh fruit, vegetable juice and carbonated soft drink consumption, particularly after brushing teeth before going to bed; and xerostomia were strong risk factors for tooth wear with high odds ratios. Brushing habits, including the use of toothbrushes with moderate bristles, monthly change of toothbrushes and brushing with horizontal pulling movements; vitamin C intake; and anorexia and bulimia were moderate risk factors with relatively low odds ratio’s. Collectively, these were the primary risk factors for tooth wear.

**Figure 5:**
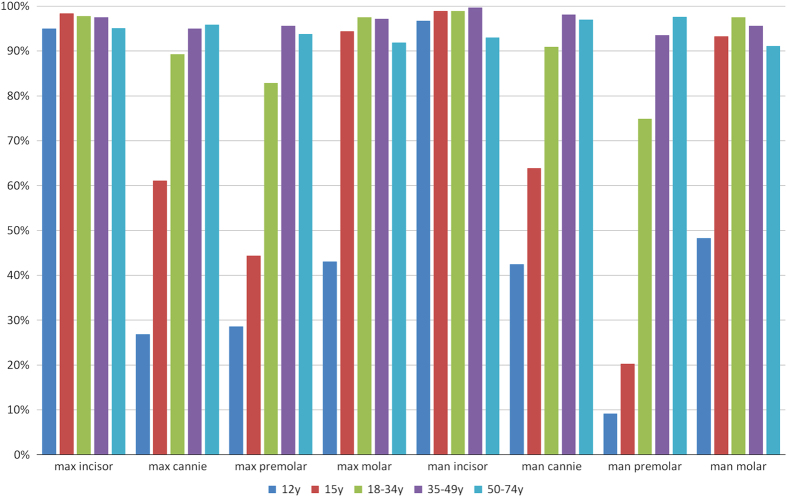
Prevalance of a BEWE score of 2–3 for the maxillary and mandibular teeth in each age group.

Among the patient-related factors, age was the strongest risk factor for wear in all tooth groups. Figure 5 shows a comparison of the odds ratios for the prevalence of wear in the maxillary and mandibular teeth in each age group. The 50–74-year-old group presents the highest prevalence of tooth wear in the maxilla canines and the mandible premolars. In the maxilla premolars, mandible incisors and mandible canines, the 35–49-year-old group presents the highest prevalence of tooth wear. However, the 18–34-year-old group has the highest prevalence in the maxilla and the mandible molars. In the maxilla incisors, the 15-year-old group shows the highest prevalence rates for tooth wear. When the entire age range of 12–74 years was considered, the maxillary and mandibular incisors showed the highest prevalence rates for tooth wear, whereas the maxillary and mandibular canines and premolars showed a significant increase in tooth wear with age.

The results of multiple logistic regression analysis showed that the consumption of fresh fruits, vegetable juice and carbonated soft drinks was significantly associated with tooth wear (Table 4). The odds ratio for fresh fruit consumption was 10.753; the consumption of fresh fruits once a day increased the probability of tooth wear by 10.753 times (95% CI, 1003.3–1225.9%). The odds ratio for carbonated soft drink consumption was 7.692 (95% CI, 712.6–778.4%); their consumption two to six times per week increased the chances of tooth wear by 669.2% relative to the chances with occasional or no consumption. The consumption of vegetable juice one time per week and one to three times per month increased the chances of tooth wear by 3.759 and 1.953 times, respectively, relative to the chances with occasional or no consumption. In particular, consumption of any of the above after brushing teeth before going to bed was a strong risk factor for tooth wear (odds ratio, 3.593; 95% CI, 1.677–7.697).

Among the patient-related factors, xerostomia was strongly associated with tooth wear, with a 10.139-fold higher risk in patients with xerostomia than in those without, particularly for the mandibular canine (Table 4).

Table 4 summarises the results for brushing habits. Tooth wear was moderately associated with the use of brushes with medium bristles, change of toothbrushes every month and brushing using horizontal pulling movements (*P*<0.05) at all ages. Moreover, these habits primarily affected the maxillary teeth. As shown in Table 4, 32.3% participants used medium-bristle toothbrushes, and they exhibited a 2.35-fold higher risk of tooth wear compared with the 51.1% who used soft-bristle toothbrushes. Horizontal pulling movements while brushing resulted in a higher risk of tooth wear compared with mixed movements. Compared with a change in toothbrushes every 3 months, a change every month increased the chances of tooth wear.

Other important risk factors included vitamin C intake and anorexia or bulimia (Table 4). In total, 28.6% of the subjects reported vitamin C intake and were at a 2.381-fold higher risk of tooth wear compared with those with no intake. Anorexic or bulimic resulted in a moderate risk of tooth wear. Area, street, sex, socioeconomic status, type of toothpaste, brushing time, chewing habits, use of aspirin and amphetamines, and use of electric toothbrush showed no influence on tooth wear.

## Discussion

This cross-sectional study analysed tooth wear data collected for five age groups and, to the best of our knowledge, is among the few studies that have used the BEWE index for an epidemiological survey of adolescents and adults. Also, this is the first study to use the BEWE index for specific tooth surfaces and to determine the prevalence of wear for different groups of teeth. The objectives of tooth wear indices, similar to those for other oral health conditions, are to classify and record the severity of events in epidemiological surveys. The BEWE index was introduced in 2008 and was designed both for the research of public dental health and for dental clinicians. Global researchers were encouraged to employ this index to conduct national epidemiological surveys of tooth wear.^10^ However, the BEWE index does not allow the specific scoring of tooth surfaces. In the present study, all four visible surfaces (buccal, cervical, lingual/palatal and occlusal/incisal) of all present teeth were scored using the BEWE index. The results revealed that tooth wear occurs on all surfaces, with the occlusal and incisal surfaces being the most frequently affected. This was consistent with the findings of other studies.^1,11^ The cervical surfaces were also susceptible to wear. The structure of the cement–enamel junction in the cervical region is relatively weak and susceptible to wear, because this is the region where masticatory stresses concentrate.^12^ In addition, the BEWE index is focused on the wear scope instead of depth; in our present study, we included an account of the wear depth.

In the present study, in both the maxillary and mandibular dentitions, the prevalence of wear was higher for the incisors than for the molars, whereas that for the molars was higher than that for the premolars and canines. Several other studies have also shown the same findings.^1,13^ The higher frequency of wear in the incisors may be attributed to the thinner enamel, the active role of incisors in joint activities and the high retention rate of incisors in older individuals.^1^ Furthermore, incisors are among the first teeth to erupt; therefore, they are subjected to wear over a longer period of time. The 50–74-year age group showed the lowest risk of wear of the maxillary and mandibular molars, probably because of the higher rate of molar loss in ageing individuals.

Among the patient-related factors, age was the strongest risk factor for wear in all tooth groups. There is some evidence that tooth wear in adolescents is a serious and notable condition.^14–16^ In our study, starting from the adolescents, tooth wear had already reached a very serious degree. Moreover, canines and premolars showed an increasing trend in tooth wear with age. Teenagers should not be ignored if we want to control tooth wear. In reality, especially in the older group, normal physiological wear is a very important cause associated with ageing.

Tooth wear is a multifactorial disease and is considered a global epidemic.^17,18^ The role of acidic foods and beverages is important in the progression of tooth wear. There is plenty of evidence from laboratory studies indicating that acidic foods and beverages with a low pH cause enamel and dentin erosion.^19–21^ In the present study, we placed more emphasis on the frequency and habit of acidic food and beverage consumption. In particular, the consumption of fresh fruits once a day, carbonated soft drinks two to six times per week, and vegetable juice once a week and one to three times a month were found to be strong risk factors. In addition, consumption of these items after brushing teeth before going to bed was a strong risk factor. After brushing, the teeth are in a state of wear, and the mere consumption of acidic juices increases tooth wear by acids.^22^ Subsequently, when the individual sleeps, saliva secretion decreases, thus decreasing the washing and dilution of the consumed acids and increasing the likelihood of tooth wear.

In the present study, the results of multiple logistic regression analysis showed that xerostomia was a strong risk factor for tooth wear. In the oral environment, the normal secretion and flow of saliva have an important effect on washing or dilution of acidic substances on the tooth surface.^20^ This action can prevent the pH of tooth surfaces from decreasing to <5.5, thus impeding the occurrence of dental erosion.^23^ In xerostomia, the salivary flow rate and buffering capacity are decreased, thus increasing the risk of tooth wear.

There was a moderate association between tooth wear and brushing habits. The mechanical friction caused by tooth brushing accelerates dental hard tissue defects.^24,25^ The use of medium-bristle toothbrushes was the most common risk factor for wear of the incisors. Changing toothbrushes once every month also increases the mechanical friction between teeth and the toothbrush. In addition, horizontal brushing movements, which is one of the most important reasons for wedge-shaped defects, was positively associated with tooth wear.

Individuals with anorexia or bulimia should exhibit a higher prevalence of tooth wear. In the present study, we found that anorexia or bulimia may contribute to the development of tooth wear. One of the main sources of intrinsic acids is the reflux of gastric contents caused by gastrointestinal diseases, including anorexia or bulimia. During digestion, the pH of gastric contents with 0.4% hydrochloric acid is ~3.8. Anorexia and bulimia reduce the pH of the oral cavity because they cause the reflux of stomach acids, thus increasing the risk of tooth wear.^26^

Another source of acidic substances is oral drugs such as vitamin C. The subjects with occasional vitamin C intake in the present study showed a 2.381-fold higher risk of tooth wear compared with subjects with no intake. Finally, area, street, sex, socioeconomic status, type of toothpaste, brushing time, chewing habits, use of aspirin and amphetamines, and use of electric toothbrush showed no significant association with tooth wear.

Considering the high prevalence of tooth wear, we can consider tooth wear as an important aspect in oral examination and make a risk assessment form based on it just like the caries risk assessment system based on this study, and according to the assessment results decide on specific preventive measures.

## Conclusion

In conclusion, the present study determined the prevalence and risk factors of tooth wear in five age groups including adults and adolescents in Beijing. Tooth wear is a serious and notable condition not only in the elderly populations but also in adolescents. In addition, there is a notable increase of tooth wear in incisors. In terms of surfaces, the occlusal, incisal and cervical surfaces are the most susceptible to wear. Specific preventive measures should be recommended for individuals reporting excessive consumption of fruits and/or acidic beverages and those with xerostomia. In particular, incisor wear should be carefully monitored in individuals of all age groups.

Tooth wear is a multifactorial disease and its related factors include dietary habits, oral hygiene, brushing habits, xerostomia, and anorexia or bulimia. Dentists ought to carry on risk assessment following several aspects, such as dietary assessment, biology assessment and systemic diseases assessment. On the basis of the analysis of assessment, we can make further planning on the three-level prevention.

## Figures and Tables

**Table 1:** Criteria for examination

*Basic erosive wear examination criteria*		*Dentin exposure*	
*Score*	*Criteria for area*	*Depth*	*Score*
0	No erosive tooth wear	Limited to enamel	0
1	Initial loss of surface texture	Limited to dentin	1
2	Distinct defect; hard tissue loss <50% of the surface area		
3	Hard tissue loss ⩾50% of the surface area		

**Table 2:** The example of a check table

		*Area*	*Depth*
BEWE score	Buccal	**–**	**–**
BEWE score	Cervical	**–**	**–**
BEWE score	Occlusal/incisal	**–**	**–**
BEWE score	Palatal/lingual	**–**	**–**
	Maxilla	17	

Abbreviation: BEWE, basic erosive wear examination.

**Table 3:** Examples of the questions

	*6*	*5*	*4*	*3*	*2*	*1*
	*⩾2 Times per day*	*1 Time per day*	*2–6 Times per week*	*1 Time per week*	*1–3 Times per month*	*Sometimes/never*
*How do you have the following food or drink*						
(1) Fresh fruit						
(2) Fruit juice						
(3) Vegetable juice						
(4) Carbonate soft drinks						

**Table 4:** The result of risk factors evaluated using a multiple logistic regression model

	*Frequency*	B	*Sig.*	*Exp (*B)	*95% CI for Exp (*B)	
					*Lower*	*Upper*
*Fresh fruit*						
Occasionally/never	80		0.001			
1 Time per day	759 (41.9%)	2.378	0	10.753	10.033	12.259
						
*Carbonated soft drinks*						
Occasionally/never	756		0.282			
2–6 Times per week	285 (15.7%)	1.157	0.013	7.692	7.126	7.784
						
*Vegetable juice*						
Occasionally/never	1,196		0.022			
1–3 Times per month	258 (14.2%)	0.669	0.04	1.953	1.032	3.695
1 Time per week	133 (7.3%)	1.324	0.006	3.759	3.104	4.678
						
*Consumption after brushing teeth before going to bed*						
Occasionally/never	1,067		0.009			
Occasionally	497 (27.4%)	1.279	0.001	3.593	1.677	7.697
						
*Xerostomia*						
No	1,522		0.07			
Yes	209 (11.5%)	2.41	0.021	11.139	1.435	86.481
						
*Toothbrush type*						
Soft bristles	926		0.074			
Medium bristles	586 (32.3%)	0.855	0.016	2.35	1.175	4.7
						
*Brushing movement*						
Vertical	430		0.036			
Horizontal	272 (15.0%)	0.876	0.04	2.401	0.931	6.195
Mixed	1,029 (56.8%)	0.746	0.02	2.108	1.124	3.955
						
*Frequency of changing toothbrushes*						
3 Months	694		0.005			
1 Month	283 (15.6%)	0.792	0.008	2.208	2.252	2.812
						
*Vitamin C intake*						
Never	974		0.094			
Occasionally	518 (28.6%)	0.867	0.005	2.381	2.229	2.773
						
*Anorexic or bulimic*						
No	1,217		0.058			
Yes	544 (30.0%)	0.658	0.037	1.931	1.04	3.587

Abbreviation: CI, confidence interval.

## References

[bib1] Liu B, Zhang M, Chen Y, Yao Y. Tooth wear in aging people: an investigation of the prevalence and the influential factors of incisal/occlusal tooth wear in northwest China. BMC Oral Health 2014; 14: 65. 24902953 10.1186/1472-6831-14-65 PMC4080580

[bib2] López-Frías FJ, Castellanos-Cosano L, Martín-González J, Llamas-Carreras JM, Segura-Egea JJ. Clinical measurement of tooth wear: tooth wear indices. J Clin Exp Dent 2012; 4: e48–e53. 24558525 10.4317/jced.50592 PMC3908810

[bib3] Bishop K, Kelleher M, Briggs P, Joshi R. Wear now? An update on the etiology of tooth wear. Quintessence Int 1997; 28: 305–313. 9452693

[bib4] Al-Omiri MK, Lamey PJ, Clifford T. Impact of tooth wear on daily living. Int J Prosthodont 2006; 19: 601–605. 17165300

[bib5] Zhang J, Du Y, Wei Z, Tai B, Jiang H, Du M. The prevalence and risk indicators of tooth wear in 12- and 15-year-old adolescents in Central China. BMC Oral Health 2015; 15: 120. 26453049 10.1186/s12903-015-0104-9 PMC4599587

[bib6] Wang P, Lin HC, Chen JH, Liang HY. The prevalence of dental erosion and associated risk factors in 12-13-year-old school children in Southern China. BMC Public Health 2010; 10: 478. 20704718 10.1186/1471-2458-10-478 PMC2927543

[bib7] Luo Y, Zeng XJ, Du MQ, Bedi R. The prevalence of dental erosion in preschool children in China. J Dent 2005; 33: 115–121. 15683892 10.1016/j.jdent.2004.08.007

[bib8] Tao D-Y, Hao G, Lu H-X, Tian Y, Feng X-P. Dental erosion among children aged 3-6 years and its associated indicators. J Public Health Dent 2015; 75: 291–297. 25952403 10.1111/jphd.12098

[bib9] Bartlett D, Ganss C, Lussi A. Basic Erosive Wear Examination (BEWE): a new scoring system for scientific and clinical needs. Clin Oral Investig 2008; 12: 65–68. 10.1007/s00784-007-0181-5 PMC2238785 18228057

[bib10] Dixon B, Sharif MO, Ahmed F, Smith AB, Seymour D, Brunton PA. Evaluation of the basic erosive wear examination (BEWE) for use in general dental practice. Br Dent J 2012; 213: E4. 22878338 10.1038/sj.bdj.2012.670

[bib11] Schierz O, Dommel S, Hirsch C, Reissmann DR. Occlusal tooth wear in the general population of Germany: Effects of age, sex, and location of teeth. J Prosthet Dent 2014; 112: 465–471. 24636759 10.1016/j.prosdent.2013.12.005

[bib12] Lussi A, Schaffner M. Progression of and risk factors for dental erosion and wedge-shaped defects over a 6-year period. Caries Res 2000; 34: 182–187. 10773637 10.1159/000016587

[bib13] Donachie MA, Walls AW. Assessment of tooth wear in an ageing population. J Dent 1995; 23: 157–164. 7782527 10.1016/0300-5712(95)93573-k

[bib14] Bartlett DW, Lussi A, West NX, Bouchard P, Sanz M, Bourgeois D. Prevalence of tooth wear on buccal and lingual surfaces and possible risk factors in young European adults. J Dent 2013; 41: 1007–1013. 24004965 10.1016/j.jdent.2013.08.018

[bib15] Salas MMS, Nascimento GG, Huysmans MC, Demarco FF. Estimated prevalence of erosive tooth wear in permanent teeth of children and adolescents: An epidemiological systematic review and meta- regression analysis. J Dent 2015; 43: 42–50. 25446243 10.1016/j.jdent.2014.10.012

[bib16] Bartlett DW, Coward PY, Nikkah C, Wilson RF. The prevalence of tooth wear in a cluster sample of adolescent schoolchildren and its relationship with potential explanatory factors. Br Dent J 1998; 184: 125–129. 9524373 10.1038/sj.bdj.4809560

[bib17] Seligman DA, Pullinger AG, Solberg WK. The prevalence of dental attrition and its association with factors of age, gender, occlusion, and TMJ symptomatology. J Dent Res 1988; 67: 1323–1333. 3049715 10.1177/00220345880670101601

[bib18] Fareed K, Johansson A, Omar R. Prevalence and severity of occlusal tooth wear in a young Saudi population. Acta Odontol Scand 1990; 48: 279–285. 2220336 10.3109/00016359009005886

[bib19] Attin T, Weiss K, Becker K, Buchalla W, Wiegand A. Impact of modified acidic soft drinks on enamel erosion. Oral Dis 2005; 11: 7–12. 10.1111/j.1601-0825.2004.01056.x 15641960

[bib20] Gedalia I, Ionat-Bendat S, Ben-Mosheh S, Shapira L. Tooth enamel softening with a cola type drink and rehardening with hard cheese or stimulated saliva in situ. J Oral Rehabil 1991; 18: 501–506. 1762023 10.1111/j.1365-2842.1991.tb00072.x

[bib21] Bartlett DW, Fares J, Shirodaria S, Chiu K, Ahmad N, Sherriff M. The association of tooth wear, diet and dietary habits in adults aged 18–30 years old. J Dent 2011; 39: 811–816. 21911033 10.1016/j.jdent.2011.08.014

[bib22] Isaksson H, Birkhed D, Wendt LK, Alm A, Nilsson M, Koch G. Prevalence of dental erosion and association with lifestyle factors in Swedish 20-year olds. Acta Odontologica Scandinavica 2014; 72: 448–457. 24286494 10.3109/00016357.2013.859727

[bib23] Moazzez R, Smith BG, Bartlett DW. Oral pH and drinking habit during ingestion of a carbonated drink in a group of adolescents with dental erosion. J Dent 2000; 28: 395–397. 10856803 10.1016/s0300-5712(00)00020-8

[bib24] Ibiyemi O, Oketade IO, Taiwo JO, Oke GA. Oral habits and tooth wear lesions among rural adult males in Nigeria. Arch Orofac Sci 2010; 5: 31–35.

[bib25] Attin T, Siegel S, Buchalla W, Lennon AM, Hannig C, Becker K. Brushing abrasion of softened and remineralised dentin: an in situ study. Caries Res 2004; 38: 62–66. 14684979 10.1159/000073922

[bib26] Kitasako Y, Sasaki Y, Takagaki T, Sadr A, Tagami J. Age-specific prevalence of erosive tooth wear by acidic diet and gastroesophageal reflux in Japan. J Dent 2015; 43: 418–423. 25684603 10.1016/j.jdent.2015.02.004

